# Small RNA activation of CDH13 expression overcome BCR-ABL1-independent imatinib-resistance and their signaling pathway studies in chronic myeloid leukemia

**DOI:** 10.1038/s41419-024-07006-9

**Published:** 2024-08-23

**Authors:** Rui Su, Ziqi Wen, Xingri Zhan, Yiling Long, Xiuyuan Wang, Chuting Li, Yubin Su, Jia Fei

**Affiliations:** 1 Department of Biochemistry and Molecular Biology, Medical College of Jinan University, Guangzhou, China; 2 Guangdong Engineering Technology Research Center of Drug Development for Small Nucleic Acids, Guangzhou, China; 3 Antisense Biopharmaceutical Technology Co Ltd, Guangzhou, China; 4 State Key Laboratory of Bioactive Molecules and Druggability Assessment, Jinan University, Guangzhou, China; 5 Department of Cell Biology & Institute of Biomedicine, National Engineering Research Center of Genetic Medicine, MOE Key Laboratory of Tumor Molecular Biology, Guangdong Provincial Key Laboratory of Bioengineering Medicine, College of Life Science and Technology, Jinan University, Guangzhou, China

**Keywords:** Drug development, Target identification

## Abstract

BCR-ABL1-independent resistance to imatinib has no effective treatment due to its complexity and diversity. We previously reported that the CDH13 oncogene was expressed at low levels in BCR-ABL1-independent resistant CML cell lines. However, its effects on CML resistant cells and mechanisms remain unknown. This study investigated the effects of saRNA-based CDH13 activation on BCR-ABL1-independent imatinib resistance in CML and its underlying mechanism, and proposes a unique treatment method to overcome imatinib resistance. Specifically, this study demonstrated that using the DSIR (Designer of Small Interfering RNA) website tool, saRNAs targeting the CDH13 promoter region were generated and validated using qPCR and western blotting. Among the predicted sequences, C2 and C3 efficiently elevated CDH13 mRNA and protein expression, as well as inhibited the relative vitality of cells and the ability to form clones. After promoting CDH13 expression in K562-IMR cells, it inhabited the NF-κB signaling pathway and induced apoptosis in imatinib-resistant CML cells. LNP-saRNA (C3) was also observed to limit the growth of K562-IMR cells in vivo. From the above, the activation of CDH13 expression by saRNA promotes cell apoptosis by inhibiting the NF-κB signaling pathway to overcome to BCR-ABL1-independent resistance to imatinib in patients with CML.

## Introduction

Chronic myeloid leukemia (CML) is a type of cancer caused by the clonal growth of hematopoietic stem cells in the bone marrow. It was one of the first diseases to be linked to specific chromosomal aberration [1], and constitutes approximately 15% of all human leukemias. Imatinib, a tyrosine kinase inhibitor (TKI), is the first approved first-line treatment for CML [2]. Although imatinib is an effective treatment for CML, patients will always develop resistance to it over time. There are two types of resistance to imatinib: BCR-ABL1-dependent resistance and BCR-ABL1-independent resistance. Many successful treatment approaches for BCR-ABL1-dependent resistance have been adopted in the clinic. Second-generation TKIs, including dasatinib, nilotinib, and bosutinib, as well as the third-generation TKI ponatinib, have made therapy more effective [3]. Nonetheless, there is no effective clinical treatment plan for BCR-ABL1-independent resistance given its complexity. This has become a major concern for researchers in recent years.

CDH13 (T-cadherin, H-cadherin) is located on chromosome 16q24.2 and is an atypical member of the cadherin family and is closely linked to clinicopathological features and the prognosis of many types of cancer [4]. Low expression of CDH13 has been observed in many cancers, including non-small cell lung cancer [5], nasopharyngeal cancer [6], esophageal cancer [7], ovarian cancer [8], CML [9, 10], acute myeloid leukemia [11], and breast cancer [12]. Lower expression of CDH13 is closely associated with the degree of methylation of its promoter. Riener et al. [13] identified genes with copy number loss on chromosome 16q in breast cancer using microarrays. The copy number loss rate was as high as 86% for CDH13. In human lung cancer cell lines, the deletion rate of CDH13 is approximately 57% [14]. Subsequently, Toyooka et al. [15] used methylation-specific PCR to corroborate that the lower expression of CDH13 gene was partially due to hypermethylation of its promoter in breast and lung cancers. Treatment with 5′-aza-2-deoxycytidine (decitabine) restored the expression of the CDH13 gene and inhibited tumor growth. Gomez et al. [11] identified the methylation status of the CDH13 promoter in 179 patients with chronic phase CML and in 52 patients with late-stage CML, and found that CDH13 was highly methylated in patients with chronic phase CML, resulting in decreased expression of this gene, which could support the clinical behavior of the disease. This suggests that the methylation status of CDH13 may be an independent predictor of the progression and prognosis of malignancy and that modulation of its expression may be a viable treatment option.

Cancer onset is an extremely complex process, and a variety of oncogenic signaling pathways are abnormally activated. In addition, the lower expression of tumor suppressor genes (TSGs) is also a potential etiology.

With the discovery and application of RNA activation (RNAa), a new approach to combat tumors has emerged. RNAa is a gene regulatory mechanism mediated by small double-stranded RNA (dsRNA), a new technological platform that increases endogenous gene transcription and recovers protein function [16]. RNA activation therapy has advantages over traditional targeted drugs and gene therapy. For example, it is easier to produce and increases the expression of endogenous genes over time. Currently, in clinical trials, the first saRNA treatment (MTL-CEBPA) targets and up-regulates the myeloid transcription factor CCAAT enhancer binding protein (CEBPA) in advanced hepatocellular cancer [17, 18]. RNAa, a novel member of the RNA family, not only provides a new platform for the study of gene function, but also shows exceptional clinical therapeutic potential [19].

The aim of the present study was to evaluate the effects of increased expression of the oncogene CDH13 following exposure to saRNAs and to explore the effects of CDH13 on BCR-ABL1-independent resistant CML cells and the related mechanisms to identify a new feasible approach to overcome resistant in CML treatment.

## Materials and methods

### Design of CDH13-saRNAs

The design of an effective saRNA is the determining factor in the success of an experiment. The following design principles were considered: (1) the 100–1000 bp DNA sequence upstream of the transcription start site (TSS) of the target gene was used for the design of the saRNA; (2) the length of the saRNA duplex was ≈21 nucleotides; (3) the GC content of saRNA is in the range of 40–60%, and the GC-rich region and CpG island in the target gene were avoided; (4) the thermodynamic stability of the 3′end of the saRNA duplex were lower than that of the 5′end; (5) The 18th and 19th bases of the saRNA sequence were “A/T”, with “A” preferred; (6) sequences with five or more consecutive nucleotides were avoided. The location of the CDH13 promoter’s GC-rich region and CpG island were checked using the UCSC website. The transcription start site (TSS) was located and 1000 bp upstream region of the TSS was used as the target sequence. CDH13-saRNA sequences were designed online using DSIR (http://biodev.extra.cea.fr/DSIR/DSIR.html) based on the design principles mentioned above.

### Generation of imatinib-resistant cells

Imatinib was introduced at a starting dose of 200 nM to the sensitive strain K562/KCL22 and then the dose was increased to 200 nM every two weeks until it reached 10 µM to develop KCL22-IMR and K562-IMR cells. Thereafter, cells were cultured in RPMI-1640 medium with 10% FBS, 1% penicillin and streptomycin, and 1 µM imatinib.

### Microfluidic preparation of saRNA-lipid nanoparticles

The components of lipid nanoparticles (LNP) were DLin-MC3-DMA, phosphatidylcholine (DSPC), cholesterol, and PEG-DMG in the following molar ratios: 50:10:38.5:1.5 and LNP:RNAa = 10:1 (wt:wt). LNP-saRNA/NC using a microfluidic procedure was prepared by Nohay Life Science (Shanghai, China). After nanoparticle synthesis was complete, the solution was blocked (40-fold) with D-PBS without calcium and magnesium by pouring the diluted solution into an ultrafiltration tube at room temperature and centrifuged at 2000 × *g* with 1–2 mL residual liquid in the ultrafiltration tube to indicate the end of centrifugation. After repeatedly washing the membrane of the ultrafiltration tube membrane with a pipette gun, we transferred the whole solution to a 0.22 μM filter membrane. The LNP-saRNA/NC obtained in the above steps was used for the subsequent animal model study.

### Human K562-IMR/luciferase-transplanted animal model

Four-week-old B-NDG mice (Beijing Bioseto Company, China) were housed at the Animal Experiment Management Centre of Jinan University. All animal experiments (20230313-07) were approved by and adhered to the relevant regulatory standards of the Institutional Animal Care and Use Committee at the Institute of Laboratory Animal Science, Jinan University (Guangzhou, China). In terms of experimental design, the 4R principle (reduction, refinement, replacement, and responsibility) was followed. After completing isolation, mice were injected via tail vein with 1 × 10^6^ K562-IMR^Luc^ cells. On day 7, in vivo imaging was performed for each B-NDG mouse. Place B-NDG mice in a cage, then randomly pick up the mice and divide them into three groups. For the Blank group (*n* = 7), K562-IMR cells that stably expressed luciferase after lentivirus infection were injected into the tail vein of mice in the blank group and no drugs were given to mice. For the LNP-NC group (*n* = 6), K562-IMR cells were injected into the tail vein, and mice were subsequently injected with LNP-NC (5 mg/kg). For the LNP-RNAa group (*n* = 7), K562-IMR cells were injected into the tail vein and LNP-RNAa (5 mg/kg) treatment was administered to mice. One treatment every 3 days, for a total of 3 treatments. In vivo imaging was used to assess the proliferation and metastasis of K562-IMR/luciferase cells in mice. The survival and body weights of the B-NDG mice were recorded and survival curves were generated for the mice in various experimental groups.

### Statistical analysis

GraphPad Prism 8 was utilized in order to perform the statistical analysis (GraphPad). The findings are presented as the mean plus the standard deviation. A one-way analysis of variance followed by the post hoc Bonferroni test was used to assess whether there were statistically significant differences between the groups. The Student’s *t*-test was utilized for paired analyses. Data are presented as the mean ± SD, obtained from at least three independent experiments. Statistical significance was determined using Student’s *t*-test versus the control group; *P* < 0.05 was considered statistically significant. The log-rank test was used to perform the analysis of the Kaplan–Meier survival curves.

## Results

### Abnormal expression of CDH13 in pancancer

**Fig. 1:**
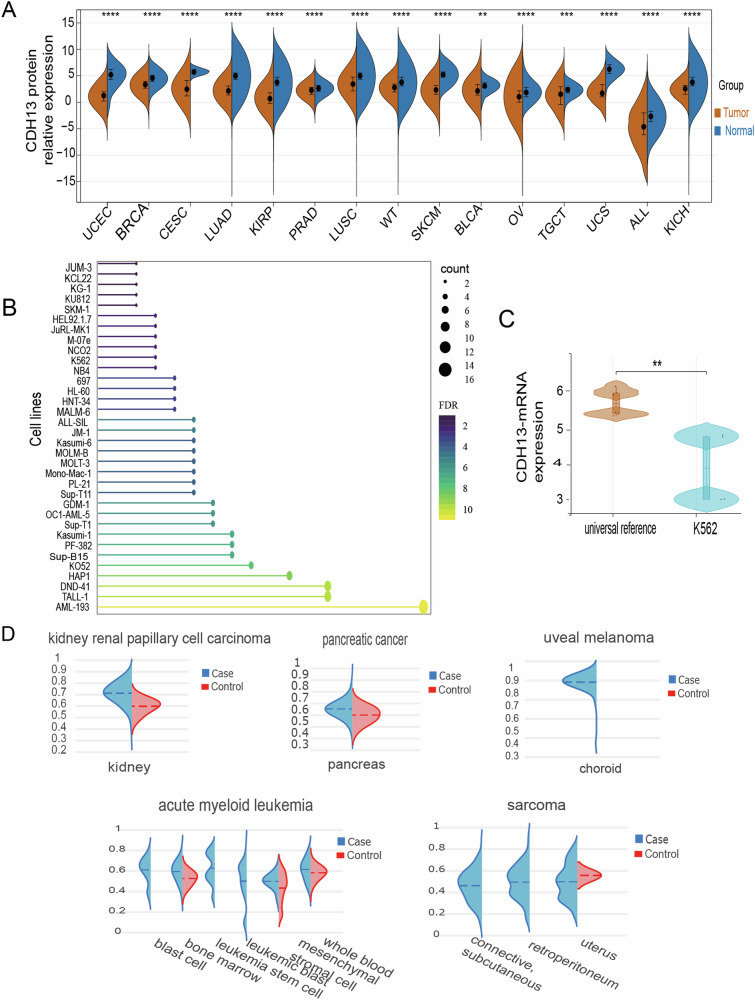
Abnormal expression of CDH13. **A** Analysis of CDH13 gene expression in datasets extracted from the UCSC and TCGA databases. **B** CDH13-mRNA expression in different hematologic tumor cell lines via the CCLE database. nTPM is the standardized transcript per million value. **C** Comparison of CDH13 mRNA expression in K562 cells and a universal reference. **D** Analysis of CDH13 gene methylation by the EWAS Data Hub. CDH13 may result in low expression in pan-carcinoma due to promoter hypermethylation.

To obtain CDH13 expression in various cancers, the uniformly normalized pancancer dataset TCGA, TARGET, GTEx, was downloaded from the UCSC database (ENSG00000140945). The dataset included uterine corpus endometrial carcinoma (UCEC), Breast invasive carcinoma (BRCA), and cervical squamous cell carcinoma and endocervical adenocarcinoma (CESC) and showed a significant down-regulation of CDH13 (Fig. 1A). To determine the level of CDH13 in various hematological tumor cell lines, datasets were obtained from the CCLE database. K562, KCL22, KU812, and NB4 had lower expression of CDH13 mRNA than other tumor cell lines of the hematological system, and were selected for subsequent experiments (Fig. 1B, C). The methylation of the CDH13 promoter has been hypothesized to be responsible for its low expression. Thus, we analyzed the methylation levels of CDH13 in several cancers using the EWAS Data Hub database (ENSG00000140945). The results reveal hypermethylation of the CDH13 promoter in cancers such as kidney renal papillary cell carcinoma (KIRP), pancreatic cancer, uveal melanoma (UM) and acute myeloid leukemia (AML) and sarcoma (Fig. 1D). CDH13 may be expressed at a low level in pancancer due to the aberrant methylation status of its promoter. Furthermore, the expression of CDH13-mRNA in the CML cell line was low among different hematologic tumor cell lines. This implies that the tumor suppressor gene CDH13 may be under-expressed in CML cell lines, which would impair biological function.

### Design and filtering of CDH13-saRNAs

**Fig. 2:**
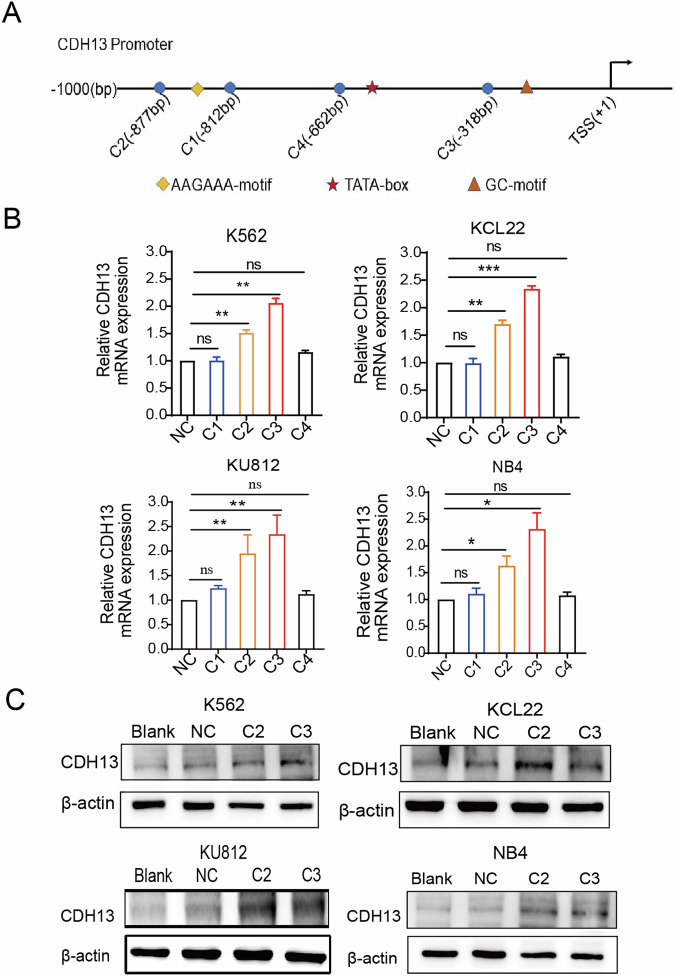
Design and validation of saRNAs to enhance CDH13 expression in CML cells. **A** CDH13-saRNA-1 (C1), CDH13-saRNA-2 (C2), CDH13-saRNA-3 (C3), and CDH13-saRNA-4 (C4) target the CDH13 promoter. CDH13 promoter region selected from the Transcription Start Site (TSS) to upstream regions of -1000 bp. The repeat elements (rhombus), TATA box (pentacle) and GC-motif (triangle) were avoided in the saRNA design. **B** saRNAs (C1–C4) were transfected into K562, KCL22, KU812, and NB4 via Lipofectamine^TM^ 2000. qPCR was used to confirm the CDH13-mRNA expression after 72 h of transfection with saRNAs (30 nM) (*P < 0.05, **P < 0.01, ***P < 0.001; saRNAs (C1–C4) compared with NC; mean ± SD; *n* = 3). **C** After transfection of saRNAs (50 nM) for 96 h, respectively, the levels of CDH13 protein expression were analyzed by western blotting. The results showed that saRNAs (C2, C3) could effectively upregulate the mRNA and protein expression of CDH13.

To explore the function of the CDH13 gene, the DSIR online software tool was used to design saRNAs targeting the CDH13 promoter based on design principles in leukemia cells. Initially, the four best-predicted sequences were filtered and named C1–C4 (Fig. 2A). To detect the minimum efficient inhibitory concentration of saRNA, we transfected various concentrations of saRNA into K562 cells and found that at a final concentration of 30 nM, saRNA effectively inhibited the relative cell viability of K562 (Fig. S1). There was no discernible change in potency as the maximum concentration increased. For subsequent experiments, maximum concentrations of 30 nM and 50 nM were selected. Among the four best-predicted sequences, compared to the NC group, C2 and C3 significantly enhanced CDH13-mRNA. In K562, KCL22, KU812, and NB4 cells, C2 activated CDH13-mRNA by 1.53, 1.75, 1.91, and 2.34 folds, respectively. In response to C3 treatment, CDH13-mRNA was elevated by 2.08, 2.37, 2.35, and 2.34 folds, respectively (Fig. 2B). Similarly, the protein expression level of CDH13 was also upregulated (Fig. 2C). In K562, KCL22, KU812, and NB4 cells, the saRNAs (C2, C3) were able to efficiently activate CDH13 expression. These results indicated that the CDH13 targeting saRNAs designed online C2 and C3 were effective sequences that successfully activated CDH13 expression.

### CDH13-saRNAs (C2, C3) suppressed the growth of CML cells

**Fig. 3:**
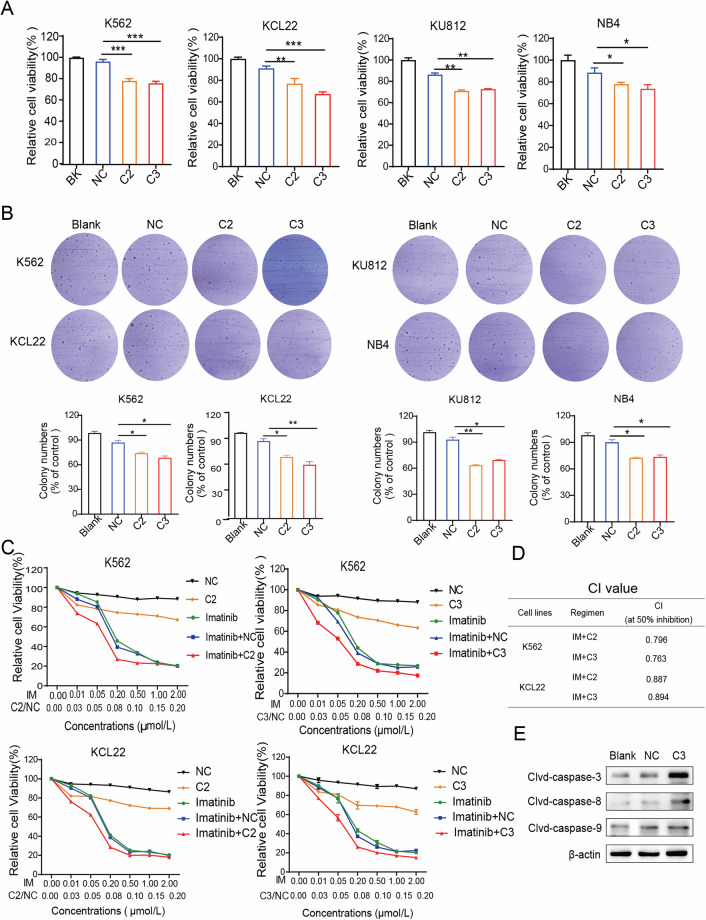
CDH13-saRNAs (C2, C3) suppressed the growth of CML cells. **A** After successfully screening saRNAs that could activate the CDH13 gene, the potency of saRNAs-CDH13 on K562, KCL22, KU812, and NB4 cells was detected. For cells transfected with saRNAs (50 nM), the relative vitality was determined via the MTT assay (NC compared with saRNAs (C2, C3); mean ± SD; *n* = 3, *P < 0.05, **P < 0.01, ***P < 0.001). **B** Soft agar clone formation assay to verify the effect of saRNAs (50 nM) on clone formation in K562, KCL22, KU812, and NB4 (**P < 0.01, ***P < 0.001 NC compared with saRNAs (C2, C3); mean ± SD; *n* = 3). **C** Imatinib (IM) was co-incubated with saRNAs in K562 and KCL22 cells, respectively. The relative viability of the cells was measured by the MTT assay after a 72 h culture. **D** The CI value of 50% inhibition for each treatment group was calculated. **E** The Blank group received no treatment and the NC and C3 groups were transfected with K562 cells at a concentration of 50 nM. After a 96 h culture, cells were collected for western blotting analysis, and proteins associated with the regulation of cell proliferation and apoptosis were identified. The result demonstrated that saRNA (C2, C3) could suppress the growth of K562, KCL22, NB4, and KU812 cells. Furthermore, when combined with imatinib, the saRNA reduced the viability of CML-sensitive cells.

After successful filtering of saRNAs that activate the CDH13 gene, the potency of saRNAs was detected in K562, KCL22, KU812, and NB4 cells. The MTT assay results that were obtained after the cell lines were exposed to the saRNAs revealed that the C2 and C3 had a significant impact on the viability of the tumor cells. Using a concentration of 30 nM of C2 or C3, the viability of the malignant cells significantly decreased to 15–25% approximately (Fig. 3A). CML is a malignant tumor formed by clonal proliferation in bone marrow hematopoietic stem cells, so the soft agarose clone formation assay can be applied to detect the change in cell proliferation ability induced by saRNAs. C2 and C3 significantly suppressed the ability of colony formation of K562, KCL22, KU812, and NB4, as evidenced by the significantly lower number of clones formed compared with the control groups (Fig. 3B). Next, we determined whether saRNAs could enhance the inhibitory influence of imatinib on CML cells. We treated K562 and KCLL cells with different concentrations of imatinib in combination with saRNA, and calculated a CI value of 50% inhibition for each treatment group based on Chou and Talalay’s median-effect analysis [20]. The results showed that the CI values of IM + C2 group and IM + C3 group of K562 cells were 0.796 and 0.763, respectively, and the CI values of IM + C2 group and IM + C3 group of KCLL2 cells were 0.887 and 0.894, respectively. They are all <1. These results suggest that saRNA (C2, C3) can synergistically inhibit CML cells with Imatinib (Fig. 3C, D). To determine how upregulation of CDH13 expression influenced CML cell growth, we performed western blotting assays to identify proteins involved in the regulation of cell proliferation and apoptosis. After transfection with C3, the expression of caspase3/8/9 was upregulated in K562 cells, which promoted their apoptosis (Fig. 3E). In contrast, the expression of proteins associated with cell proliferation (CDK2, CDK4, CDK6, p21, p27, and CyclinD1) was not significantly altered (Fig. S2). SaRNA (C2, C3) inhibited the growth of K562, KCL22, KU812, and NB4 cells and synergistically inhibits the viability of CML-sensitive cells with imatinib. Furthermore, CDH13 activation in K562 cells promoted cell apoptosis by upregulating the expression of apoptosis-related proteins caspase3/8/9.

### CDH13-saRNAs overcame resistance to BCR-ABL1-independent imatinib by inhibiting the NF-κB signaling pathway

**Fig. 4:**
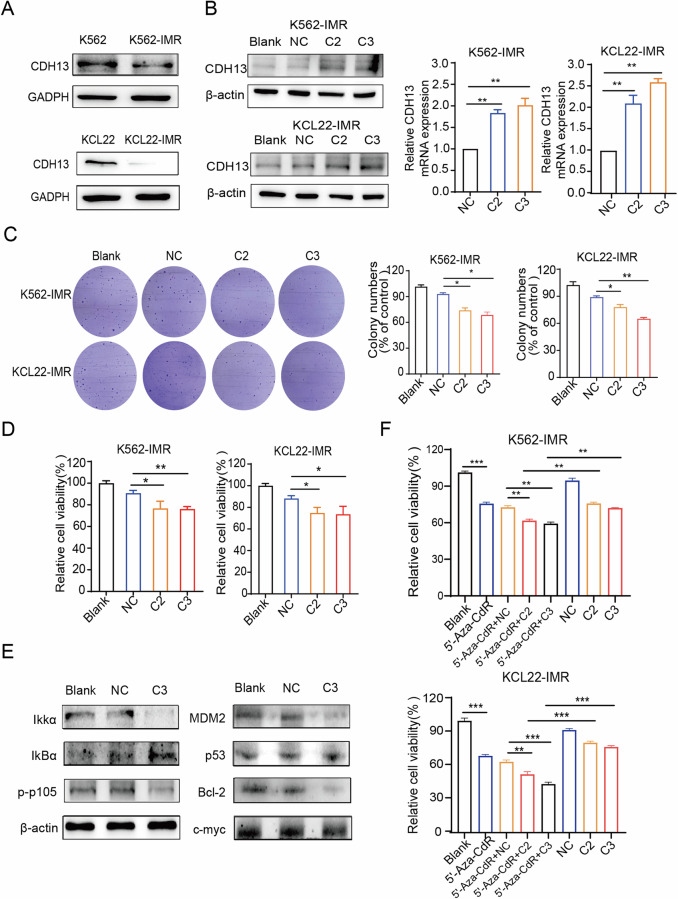
CDH13-saRNAs overcome BCR-ABL1- independent imatinib resistance by inhibiting the NF-κB signaling. **A** Differences in expression of CDH13 protein in K562, KCL22-sensitive, and K562-IMR, KCL22-IMR-resistant cells by western blotting. **B** saRNAs (50 nM) were transfected into K562-IMR and KCL22-IMR cells, respectively. qPCR was used to confirm the CDH13-mRNA expression levels after 72 h of transfection with saRNAs (30 nM) (**P < 0.01, saRNAs (C2, C3) versus NC; mean ± SD; *n* = 3). Western blotting confirming the CDH13-protein expression levels after 96 h of transfection with saRNAs (50 nM). **C** The soft agar clone formation assay verified the effects of saRNAs on clone formation in K562-IMR and KCL22-IMR cells (*P < 0.05, NC compared with C2, C3; mean ± SD; *n* = 3). **D** saRNAs were transfected into K562-IMR and KCL22-IMR cells, and the relative viability was measured by MTT after a 72 h culture (*P < 0.05, **P < 0.01, NC compared with C2, C3; mean ± SD; *n* = 3). **E** Western blotting was used to detect the regulation of apoptosis-related proteins in K562-IMR cells. **F** 5′-Aza-CdR (5 μM), C2(50 nM), C3(50 nM) and 5′-Aza-CdR combined with NC (50 nM), C2 (50 nM) and C3 (50 nM) treated imatinib-resistant CML cells. The relative viability of the cells was measured by the MTT after a 72 h culture (**P < 0.01, ***P < 0.001, mean ± SD; *n* = 3). The above experiments showed that activation of CDH13 induced apoptosis by inhibiting the NF-κB signaling pathway in CML-resistant cells. Decitabine combined with saRNA(C2, C3) synergistically inhibited the relative viability of CML-resistant cells.

A correlation was observed demonstrated between silenced genes due to aberrant methylation and drug resistance. To do so, we generated CML strains resistant of imatinib. K562 and KCL22 were treated with increasing imatinib concentrations and their resistance was determined using the MTT assay. The results showed that K562-IMR and KCL22-IMR strains became resistant to when the IC_50_ value increased to 5.568 μM, 5.289 μM, separately (Fig. S3). Subsequently, we compared CDH13 expression between CML-sensitive and imatinib-resistant cell lines using western blotting. Of note, K562-IMR/KCL22-IMR had significantly lower expression of CDH13 than K562/KCL22 (Fig. 4A). To restore the biological function of CDH13, C2 and C3 were transfected into K562-IMR and KCL22-IMR cells, respectively. Exposure to saRNAs (C2, C3) upregulated CDH13-mRNA and protein expression levels in K562-IMR and KCL22-IMR cells (Fig. 4B). Using the MTT and soft agar colony formation assay, the saRNAs (C2, C3) inhibited cell viability and clone formation ability (Fig. 4C, D). Therefore, saRNAs (C2, C3) also inhibit the growth of K562-IMR and KCL22-IMR cells.

Previous research discovered that CDH13-saRNA transfection in K562 promoted apoptosis. Encouraged by these results, we proceeded to examine whether this phenomenon is also present in imatinib-resistant cells. The transfection of saRNA (C2, C3) promoted the apoptotic process in K562-IMR and KCL22-IMR cells as assessed by flow cytometry (Fig. S4). To elucidate the specific signaling pathways that regulated apoptosis in imatinib-resistant CML cells after activation of CDH13, we tested the changes in expression of key proteins from the SAPK/JNK, TRAIL/FADD, and NF-κB signaling pathways using western blotting (Fig. S5). Upregulation of CDH13 expression by saRNAs (C2, C3) in K562-IMR cells was accompanied by downregulation of IKKα, upregulation of IkBα, and inhibition of p105 phosphorylation. Furthermore, saRNAs (C2, C3) regulated NF-κB downstream genes and suppressed the expression of the oncogenes MDM2, c-myc, and Bcl-2 (Fig. 4E). Activation of CDH13 in K562-IMR cells inhibited the NF-κB signaling pathway to promote apoptosis. Low expression of CDH13 in drug-resistant strains was associated with a higher level of methylation of this promoter (Fig. S6A). We then tested whether the enhancement of CDH13 expression by CDH13-saRNA in CML cells was due to the lower methylation status if its promoter. The results demonstrated that CDH13-saRNA-induced expression was not related to promoter demethylation (Fig. S6B). Although saRNA is incapable of regulating methylation, we found that saRNA was processed into imatinib-resistant cells with decitabine and coordinately inhibited cell viability (Fig. 4F). The above results indicate that CDH13 promoted apoptosis by inhibiting the NF-κB signaling pathway, thus overcoming resistance to BCR-ABL1-independent drugs. Furthermore, decitabine combined with saRNA(C2, C3) synergistically inhibited the relative viability of CML-resistant cells.

### CDH13-saRNA inhibited the growth of K562-IMR cells in vivo

**Fig. 5:**
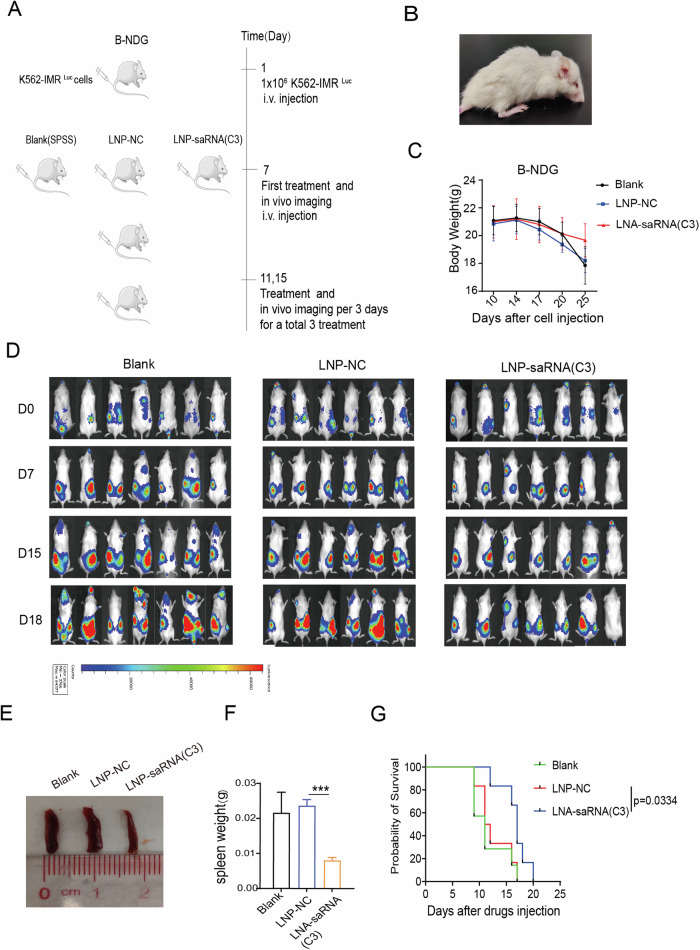
CDH13-saRNA inhibits the growth of K562-IMR in vivo. **A** A BCR-ABL1-independent imatinib-resistant CML xenograft mouse model was used to detect the potency of LNP-saRNA (C3). **B** Arch dorsal morphology of onset mice. **C** Body weight change of B-NDG mice. **D** Live animal imaging showing proliferation and spread of K562-IMR/luciferase cells in B-NDG mice. The darker color indicates higher number of cells in vivo. **E** Spleen size change of B-NDG mice. **F** Spleen weight comparison of B-NDG mice (***P < 0.001, LNP-NC compared with LNP-saRNA(C3); mean ± SD; *n* = 6). **G** Survival curve of B-NDG mice. CDH13-saRNA inhibited the growth of K562-IMR cells in B-NDG mice.

Encouraged by CDH13 suppression for imatinib-resistant cells in vitro, we next investigated their therapeutic efficacy in vivo using a BCR-ABL1 independent resistant CML xenograft model. Drugs were administered every three days for a total of three times (Fig. 5A). During feeding, we observed that the early-treated mice had unruly fur, were easily scared and exhibited pronounced back arching (Fig. 5B). Mice in the LNP-saRNA group had lost substantially less weight compared to those of the Blank and LNP-NC groups (Fig. 5C). After in vivo imaging, dosing was performed several D0 days on this day. Live imaging of mice was used to monitor the proliferation and localization of K562-IMR cells. The fluorescence intensity was lower in B-NDG mice injected with LNP- saRNA, which inhibited tumor cell spread when compared to the NC group on D15 and D18 (Fig. 5D). After dissecting the mice, we collected their spleens and discovered that the spleens of the mice in the NC group were substantially enlarged, while the enlargement of the spleen in the LNP-saRNA group was significantly reduced (Fig. 5E, F) and their survival time was significantly increased (Fig. 5G). Together, these data demonstrate that CDH13-saRNA inhibits the growth of K562-IMR cells in B-NDG mice.

### RNAa activate CDH13 expression to overcome BCR-ABL1-independent imatinib resistance

**Fig. 6:**
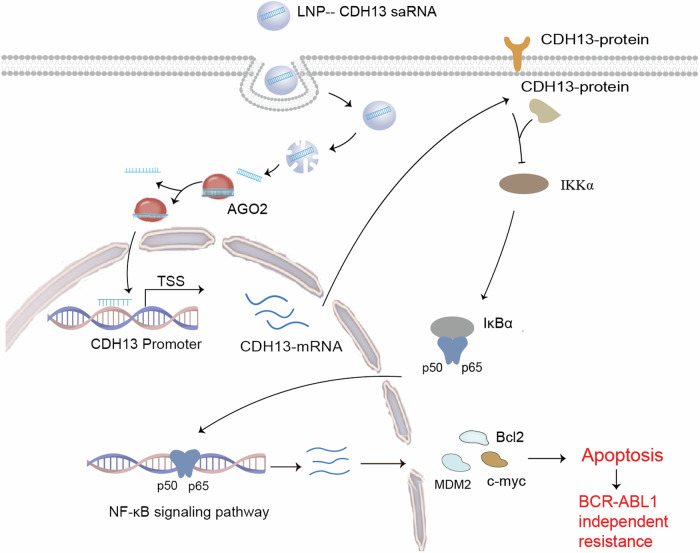
Schematic diagram of the action mechanism of CDH13-saRNA in BCR-ABL1 independent imatinib resistance. Activation of saRNA based on saRNA inhibited the expression of the NF-κB signaling pathway to promote apoptosis and overcome BCR-ABL1-independent imatinib resistance.

In general, we used RNAa technology activate CDH13 expression to inhibited the expression of the NF-κB signaling pathway for promoting apoptosis and overcome BCR-ABL1-independent imatinib resistance (Fig. 6). RNAa-based drugs have many promising advantages for the treatment of a broad range of diseases because they can increase gene expression and target genes that are clinically under-expressed or that could not have been previously treated with drugs. In the future, the use of RNAa alone or in combination with chemotherapeutic agents could also be a new and powerful approach to treat diseases caused by low gene expression.

## Discussion

Imatinib is the first-line therapeutic drug for patients with primary CML, and despite its groundbreaking therapeutic efficacy, more than 25% of patients develop resistance to therapy. Currently, there are no clinically available therapeutic regimens that target BCR-ABL1-independent resistance. Therefore, we can develop new therapeutic strategies based on various mechanisms or combine them with existing clinical strategies to improve the efficacy of treatments for imatinib-resistant CML. CDH13 is highly methylated in cancers such as CML [11], non-small cell lung cancer [21], and breast cancer [22], resulting in a significant decrease in its expression level and the loss of biological functions of oncogenes (Fig. 1A). Most significantly, we found lower expression in imatinib-resistant CML cells than in sensitive CML cells. Therefore, we propose that imatinib resistance may be related to the low level of CDH13 expression. In this study, we used a saRNA strategy to increase CDH13 expression to restore its biological function in imatinib-resistant CML cells. Furthermore, we explored the molecular mechanism involved in inhibition of CDH13 in imatinib-resistant CML cell growth, providing a new strategy to overcome resistance to imatinib-independent BCR-ABL1.

SaRNA-based gene activation is a novel approach to study how genes work and may be used to treat diseases caused by low expression of tumor suppressor genes (TSG). We designed saRNA targeting the CDH13 promoter using DSIR online tools (Fig. 2A). Wu et al. [23] designed saRNAs targeting p21 and were able to increase p21 mRNA expression from 3.77 to 5.03-fold. In this study, we transfected saRNA and found that the level of CDH13 mRNA increased 1.53- to 2.37-fold (Fig. 2B). The difference in the effect of induction was possibly due to the complexity of the target gene promoter elements, the type of cell, or the efficiency of transfection. After the saRNAs successfully increased CDH13 expression, we found that the relative activity and clone formation ability of K562, KCL22, KU812, and NB4 could be suppressed (Fig. 3A, B). Several studies have shown that CDH13 inhibits cancer cells [24]. Co-administration of cytotoxic drugs that work through different mechanisms may improve their synergistic effects. Several studies have shown that CML cells are more sensitive to imatinib when combined with trichothecene [25], platinum-based anticancer drugs [26], or quercetin [27]. This study found that saRNA can synergistically inhibit the growth of CML cells with imatinib based on Chou and Talalay’s median-effect analysis. (Fig. 3C, D). Subsequently, inducing CDH13 expression was found to increase the expression of CDH13 caspase3/8/9 and increased the expression of caspase3/8/9 to promote K562 cell apoptosis (Fig. 3E).

Many experts agree with the view that epigenome changes are closely associated with the development of clinical resistance. For example, the number of imatinib-resistant patients with methylation of organic solute carrier partner 1 (OSCP1) is much higher than the number of imatinib-sensitive patients with OSCP1 methylation. Silencing this gene prevents imatinib from being transported to cells [28], leading to the development of resistance to imatinib. In this study, K562-IMR and KCL22-IMR were developed by the gradually increasing exposure to higher imatinib concentrations (Fig. S3). The bisulfite experiment showed that K562-IMR and KCL22-IMR cells were hypermethylated in CDH13 compared to the matching sensitive cell lines (Fig. S6A). This resulted in K562-IMR and KCL22-IMR having lower expression than wild-type K562 and KCL22. Cells resistant to imatinib were then treated with saRNA (C2, C3), which successfully induced the expression of CDH13 (Fig. 3B). The MTT and colony assays demonstrated that the saRNAs (C2, C3) were able to inhibit relative cell viability and proliferation ability (Fig. 3C, D).

BCR-ABL1-independent resistance to imatinib can also be caused by activation of an abnormal signaling pathway. NF-κB signaling activity is activated in BCR-ABL1-independent imatinib-resistant cells in CML. This is because Ser177/Ser181 on IKK and Ser536 on p65 NF-κB have become more phosphorylated [29]. After NF-κB signaling is activated, upregulation of the apoptosis gene inhibitor allows CML cells to avoid the apoptosis effects of chemotherapeutic drugs and induce drug resistance. Currently, there are inhibitors of this pathway, such as PS1145 and AS602868 [30]. Blocking NF-κB can cause AML cells to be more susceptible to death by apoptosis [31]. SaRNAs were developed that could induce cells apoptosis as determined by flow cytometry (Fig. S4). To investigate the relative mechanism of CDH13 that regulates apoptosis, we performed a screening assays to detect changes in the expression of key proteins in the SAPK/JNK, TRAIL/FADD, and NF-κB signaling pathways by western blotting. The results showed that activating CDH13 regulated the expression of IKK, IkBα, and their downstream genes c-myc, Bcl2, and MDM2 (Fig. 3E). In conclusion, activating CDH13 suppressed the NF-κB signaling pathway that induced cells to apoptosis, making BCR-ABL1 independent CML cells more susceptible to imatinib. Although saRNA cannot regulate methylation (Fig. S6B), we found that exposure of saRNA to an imatinib-resistant cell treated with decitabine, could coordinately inhibit cell proliferation(Fig. 4F). However, decitabine changes the methylation patterns of the entire genome, and not only that of the target gene, which can cause unwanted side effects. Therefore, a targeted saRNA strategy able to restore the expression of target genes might be safer in these cases. The effects of saCDH13 on tumorigenesis was evaluated using a BCR-ABL1-independent imatinib-resistant CML xenograft animal model by injecting K562-IMR/luciferase cells into B-NDG mice. In this study, we used an LNP delivery method to allow saRNA to work better. The results of the in vivo experiments showed that, compared to the LNP-NC group, the fluorescence intensity of B-NDG mice injected with LNP-saRNA (C3) was lower. The spread of tumor cells in mice was also delayed and survival of mice was much longer (Fig. 5D, G).

### Supplementary information

SUPPLEMENTAL MATERIAL

Original Data

## Data Availability

All data associated with this study are presented in the paper or the Supplementary Data. Materials that support the findings of this study are available from the corresponding author upon request.
