# Supplementary material for: Small RNA activation of CDH13 expression overcome BCR-ABL1-independent imatinib-resistance and their signaling pathway studies in chronic myeloid leukemia
Source: Cell Death Dis. 2024 Aug 23;15(8):615. doi: 10.1038/s41419-024-07006-9 (PMC11343752; doi:10.1038/s41419-024-07006-9)
Supplement: Supplementary file 1 — SUPPLEMENTAL MATERIAL [file 41419_2024_7006_MOESM1_ESM.docx]

**Supplemental Material**

**Supplemental table**

Table 1. The sequences of primers and saRNAs

| Target gene | 5‘-3‘ | Sequence |
| --- | --- | --- |
| CDH13 | FW | 5'-CCCAGGTGCTGCTGCTAA-3' |
|  | RW | 5'-CTGGCTGATTGATATGGAAC-3' |
| GAPDH | FW | 5'-CAATGACCCCTTCATTGACC-3' |
|  | RW | 5'-GACAAGCTTCCCGTTCTCAG-3' |
| saRNA-C1 | SS | 5'-GCAUGGAAAUGAUACAGUA[dT][dT] -3' |
|  | AS | 5'-UACUGUAUCAUUUCCAUGC[dT][dT] -3' |
| saRNA-C2 | SS | 5'-CAUAAAUUCUAUUGCACAA[dT][dT] -3' |
|  | AS | 5'-UUGUGCAAUAGAAUUUAUG[dT][dT] -3' |
| saRNA-C3 | SS | 5'-GGAAAUAGAGUGGAUCUUA[dT][dT] -3' |
|  | AS | 5'-UAAGAUCCACUCUAUUUCC[dT][dT] -3' |
| saRNA-C4 | SS | 5'-GGUAGUUCAGAGCUuCUGA[dT][dT] -3' |
|  | AS | 5'-UCAGAAGCUCUGAACUACC[dT][dT] -3' |
| CDH13 | UM-FW | 5'-TTAAAGAAGTAAATGGGATGTTATTTTT -3' |
|  | UM-RW | 5'-ACCAAAACCAATAACTTTACAAAACA-3' |
|  | M-FW | 5'-TAAAGAAGTAAATGGGATGTTATTTTC-3' |
|  | M-RW | 5'-AAAACCAATAACTTTACAAAACGAA-3' |

**Supplemental methods**

**Bioinformatic analyses**

The uniformly standardized pan-cancer dataset, TCGA TARGET GTEx (PANCAN, N=19131, G=60499) was downloaded from the UCSC (https://xenabrowser.net/) database, from which the expression data of the CDH13 gene was extracted for the samples. Subsequently, the CCLE database (https://portals.broadinstitute.org/ccle/about) was used to obtain CDH13-mRNA expression levels in various cell lines of leukemia. In addition, the EWAS Data Hub database (https://ngdc.cncb.ac.cn/ewas/datahub) database was used to determine the methylation levels of CDH13 in several cancers.

**Cell Culture**

Cell lines (K562, KCL22, KU812, and NB4) were cultured in RPMI-1640 medium with 10% fetal bovine serum (Gibco, USA) and 1% penicillin and streptomycin (Gibco, USA) under a 5% CO_2_ atmosphere at 37°C. K562, KU812, and NB4 cells were obtained from the Institute of Shanghai Cell Biology, China. The KCL22 cell line was kind gift from Professor Markus Muschen, Children’s Hospital of Los Angeles, CA, USA. K562-IMR and KCL22-IMR cells were developed in our laboratory. Cells were subcultured every 2–4 days, and cells in the logarithmic growth phase were selected for study.

**MTT assay**

Briefly, cells were seeded at a density of 4x10^4^ cells/mL in 96-well plates (100 μL/well) and treated with saRNA or NC. After 72 h, 20 μL MTT (5 mg/mL) was added to each well and incubated for 4 h. Subsequently, plates were centrifuged at 1200 rpm for 3 min. Each well received 150 μL DMSO after carefully aspirating the supernatant. Cell viability was assessed by measuring absorbance at 570 nm on a Bio-Tek microtiter plate reader. The proliferation inhibition rate was calculated as [1-(experimental group/control group)] x100% after three repetitions.

**Soft agar colony formation assay**

In the logarithmic growth phase, K562, KCL22, KU812, NB4, K562-IMR, and KCL22-IMR cells were harvested to detect proliferation capacity by the soft agar colony formation assay. After transfection, RPMI-1640 medium containing 20% FBS was mixed with soft agar (Sigma, USA) containing 1.2% in a 1:1 ratio and 2 mL of the mixture was added to each well of the six-well plate to form the lowest gel layer and allowed to solidify. After transfection, RPMI-1640 containing saRNAs was gently combined with 0.3% soft agar on the upper gel. Next, 1 mL was added per well to the bottom gel that had solidified while waiting for the upper gel to solidify. After observing more than 40 cell clonal groups under a microscope, 4% paraformaldehyde was added for 30 minutes. After washing with PBS, 0.6 mL of 0.005% crystal violet solution was added to each well for staining for 1 h to 2 h, and then the staining solution was aspirated and discarded. The cell clusters were washed with PBS until they were visible. Subsequently, photographs of the clusters were obtained, and image J and GraphPad were used to determine the number of cell colonies.

**Transfection of saRNA drugs**

SaRNAs (1-4) and NC (negative control, random sequences) were transfected into K562, KCL22, KU812, NB4, K562-IMR, and KCL22-IMR cells by Lipofectamine^TM^ 2000 reagent (Invitrogen, China). The final concentrations of saRNA and NC ranged 30–50 nM. For transfection, Lipofectamine^TM^ 2000 reagent was diluted with Opti-MEM and incubated for 5 minutes at room temperature. The saRNAs were diluted in Opti-MEM. After a 5 minute incubation, the Lipofectamine^TM^ 2000 reagent was gently mixed in a 1:1 (v/v) ratio with nucleic acid and incubated for 15 min at room temperature. After six hours at 37°C, the wells were replaced with complete medium RPMI-1640 (10% FBS by volume).

**Quantitative real-time**

To verify the expression of CDH13 mRNA in K562, KCL22, KU812, NB4, and K562-IMR, and KCL22-IMR cells, Trizol (Invitrogen, USA) was used as the extraction method to obtain total RNA from cells. The total RNA (500 ng) was then reverse transcribed using a SuperScript IV first-strand synthesis system (Invitrogen, USA). Relative expression of mRNA was detected by 2x SYBR Green qPCR Master Mix (Bimake, USA) according to the manufacturer's instructions by quantitative real-time PCR (qRT-PCR). The 2^-ΔΔCt^ method was used to determine the relative expression of mRNA.

**Western blotting analysis**

Following cell lysis with PMSF-containing RIPA buffer (Roche, USA) for 30 minutes. The BCA Protein Assay Kit was utilized to determine total protein concentrations (KeyGen Biotech, China). SDS-PAGE was used to separate a total of 30 µg of protein mixture from each sample, and the separated proteins were then transferred to PVDF membranes (Merck Millipore, Germany). The PVDF membrane was blocked with 5% milk (in PBST) for one hour and then incubated overnight at 4°C with primary antibody against H-Cadherin (ab167407, Abcam). As a control, antibodies against beta-actin (ab8227,Abcam) and GAPDH (ab128915, Abcam) were utilized. Cleaved Caspase-3 (#9664S, CST), Cleaved Caspase-8 (#8592S, CST), Cleaved Caspase-9 (#7237S, CST), CDK4 (#12790S, CST). IKKα（#2682S, CST）, IKBα（# 4812S, CST）, MDM2 (# 86934S, CST), Bcl-2 (#3498S, CST), c-myc (#5605S, CST). Signals were detected using HRP-conjugated anti-rabbit IgG antibodies (Cell Signaling Technology, USA), and were observed using ECL detection. Both reagents were purchased from Cell Signaling Technology.

**Combination studies**

Using the combination index (CI) method, which is based on Chou and Talalay's median-effect analysis, the effects of the combination were determined for each experimental condition. The following formula defines the confidence interval: CI is equal to (D)1/(Dx)1 + (D)2/(Dx)2, where (Dx)1 and (Dx)2 are the combined concentrations needed to create an inhibition rate, and (Dx)1 and (Dx)2 are the individual drug concentrations needed to create an inhibition rate. When two medications have similar modes of action, α = 0 indicates mutual exclusion; however, α = 1 indicates mutual non-exclusion (i.e., independent modes of action). CI > 1, CI = 1, and CI < 1 denote antagonism, additivity, and synergy, in that order.

**DNA Methylation Assay**

Samples were bisulfite-converted with the EZ DNA MethylationTM Kit (Zymo Research, USA). Methylation-specific PCR (MSP) uses amplification to determine the methylation status of a particular CpG site. Differential amplification of the template with (M) methylated and (U) unmethylated primer sets is crucial to the success of this method. MSP primers were constructed using the online platform available at <https://zymoresearch.eu/pages/services>.

**Flow cytometric detection of apoptosis**

Equal numbers of cells were placed in flow cytometry tubes and washed with PBS by centrifugation at 800 rpm for 3 min. Each group of cells were resuspended with 300 μL of Binding Buffer. Next. 3 μL of Annexin V-FITC, were added and the solution was mixed gently with a pipette, and then incubate it at room temperature for 15 minutes while shielding it from the light. After carefully adding 3 μL of the Propidium staining solution, the contents of the two vials were combined using a pipette, and then incubated at room temperature for 15 minutes while shielding it from the light.

**Supplemental results**

**Figure S1.** Different concentrations of NC and saRNA (C3) were transfected into K562 cells, and after 72 hours, the relative viability of the cells was detected using the MTT assay（***P<0.001; mean±SD; n=3）. saRNA(C3) shows a cell-inhibiting effect at 30 nM, but the efficacy does not change significantly as the concentration increases.


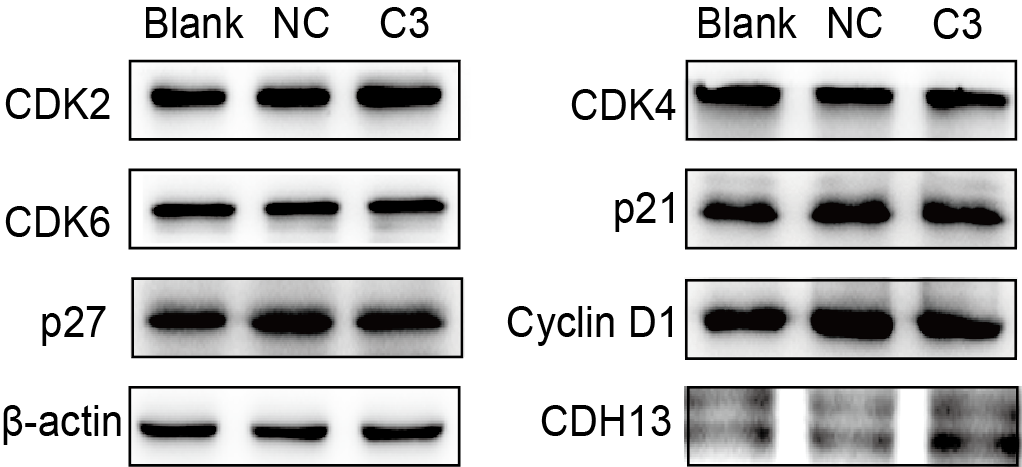


**Figure S2.** NC and saRNA(C3) were transfected into K562 cells respectively, and the expression of proteins related to the regulation of cell proliferation was examined using western blotting assays. Activation of CDH13 in K562 cells did not affect the expression of cell proliferation-related proteins.


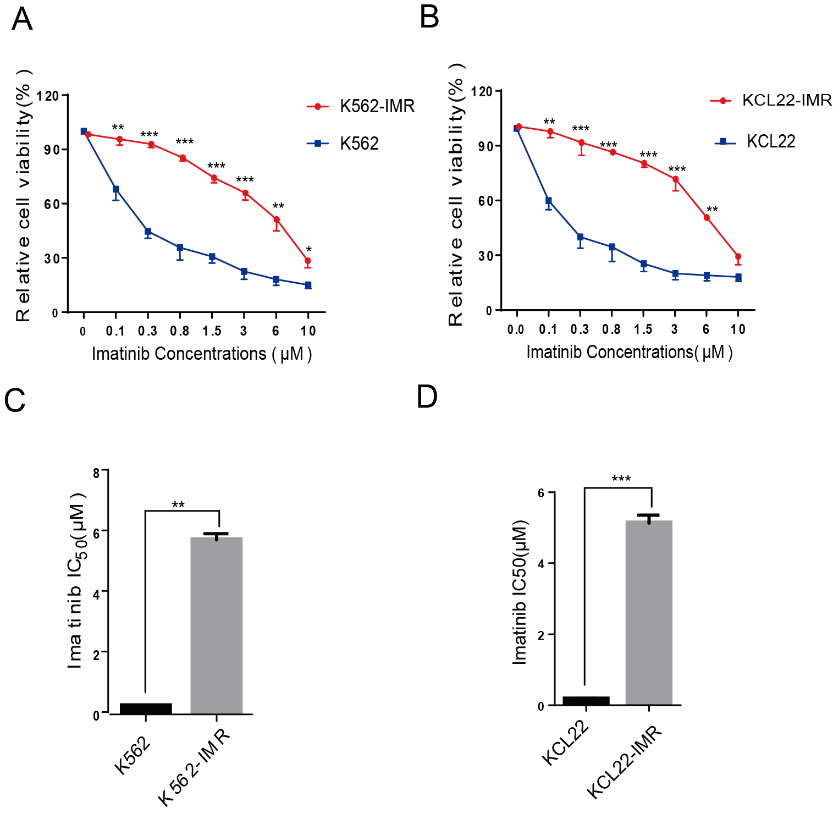


**Figure S3.** The mutation sites of BCR-ABL1 in K562-IMR were analyzed and sequenced. There were no commonly known mutations of BCR-ABL1 such as T315I in the cell lines, indicating that CML resistant cell lines are imatinib resistant strains independent of BCR-ABL1.


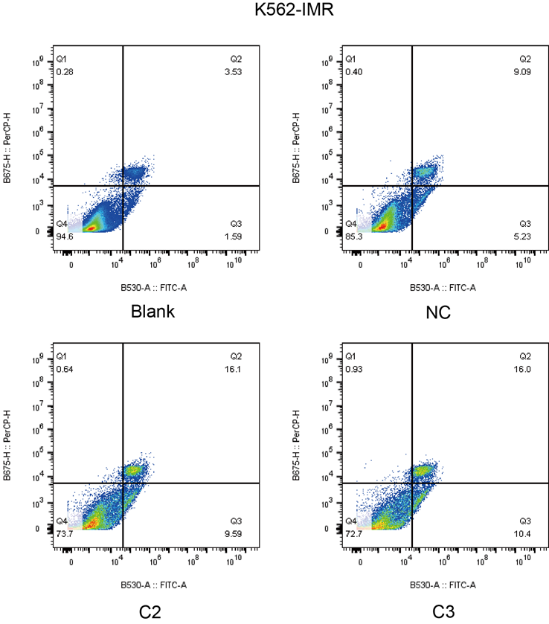

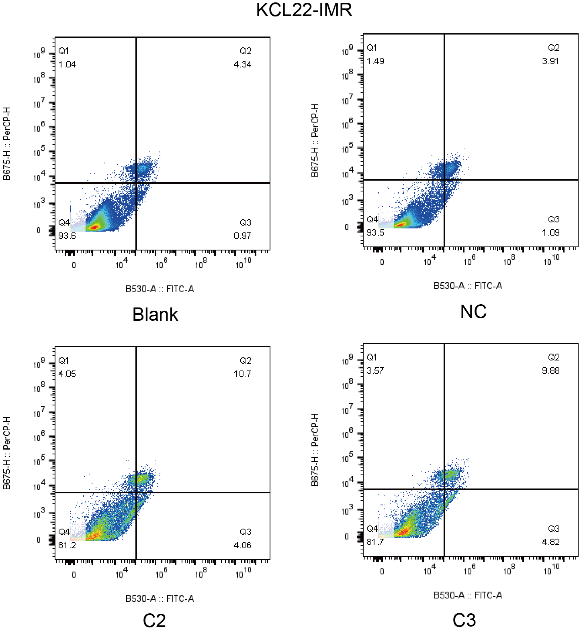


**Figure S4.** The proportion of cells with early versus late apoptosis was detected by flow cytometry after saRNA (C2,C3) transfection into K562-IMR and KCL22-IMR cells. After saRNA (C2,C3) activates CDH13 and promotes apoptosis in K562-IMR and KCL22-IMR cells.


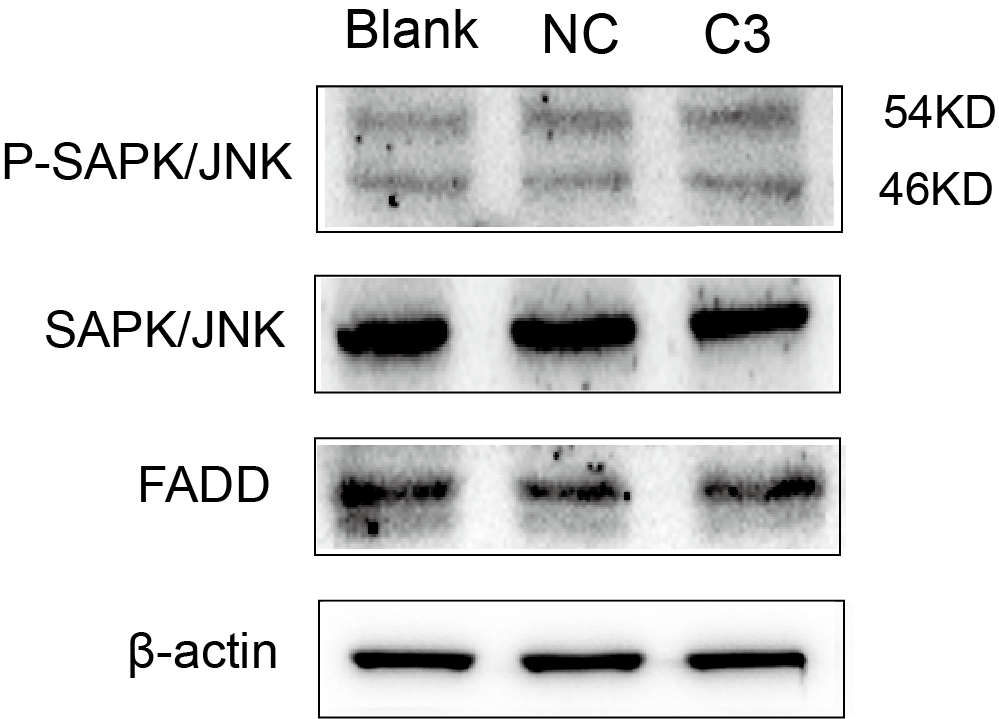


**Figure S5.** NC and saRNA (C3) were transfected into K562-IMR cells, respectively. The expression of SAPK/JNK and FADD showed no changes in expression using the western Blotting assay.


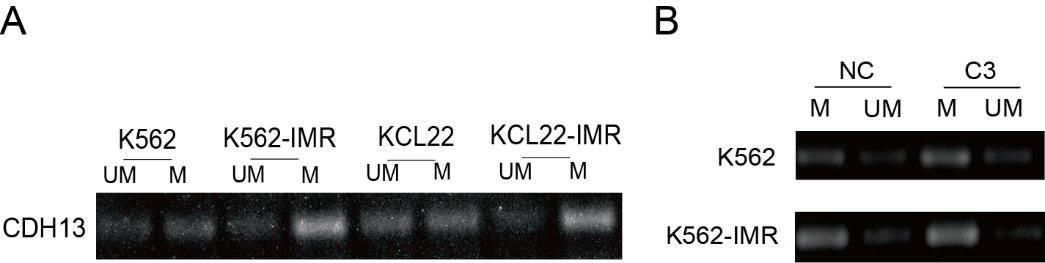


**Figure S6.** (A) K562, K562-IMR and KCL, KCL22-IMR were subjected to bisulfite to amplify methylated and unmethylated sites on the CDH13 promoter by MSP primers. (B) SaRNA (50 nM) were transfected into K562 and K562-IMR cells and the cells were colleved after a 72 h treatment. The bisulfite assay was performed, followed by PCR amplification of methylated and unmethylated sites on the promoter. UM: unmethylated site; M: methylated site.


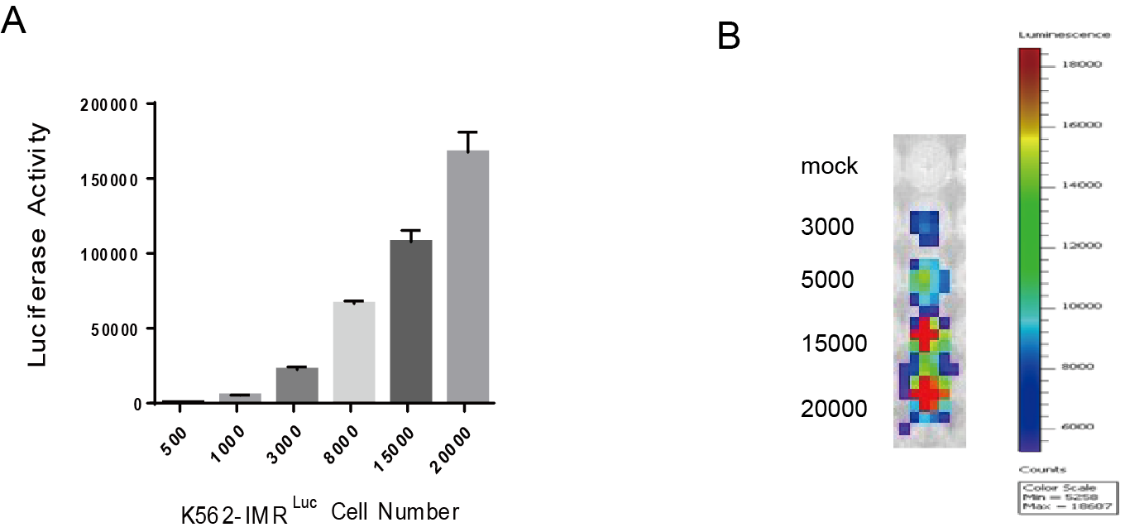


**Figure S7.** Identification of K562-IMR/luciferase cells. (A) Enzyme marker showing luciferase activity proportional to cell number. (B) Small animal live imaging system showing fluorescence intensity proportional to cell number. The results showed that K562-IMR^Luc^ cells were successfully constructed.


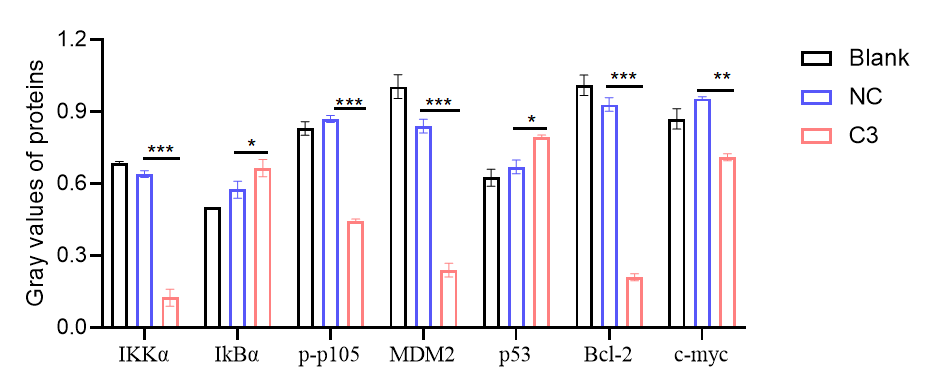


**Figure S8**. The Western blot data（Figure 4E）analyzed by gray value. The data are presented as the mean ± SD, obtained from at least three independent experiments. Significance was determined by Student’s t-test, *P < 0.05,**P < 0.01,***P < 0.01 versus the NC group.
