# Supplementary figures and images for: Small RNA activation of CDH13 expression overcome BCR-ABL1-independent imatinib-resistance and their signaling pathway studies in chronic myeloid leukemia
Source: Cell Death Dis. 2024 Aug 23;15(8):615. doi: 10.1038/s41419-024-07006-9 (PMC11343752; doi:10.1038/s41419-024-07006-9)

Figure 2C

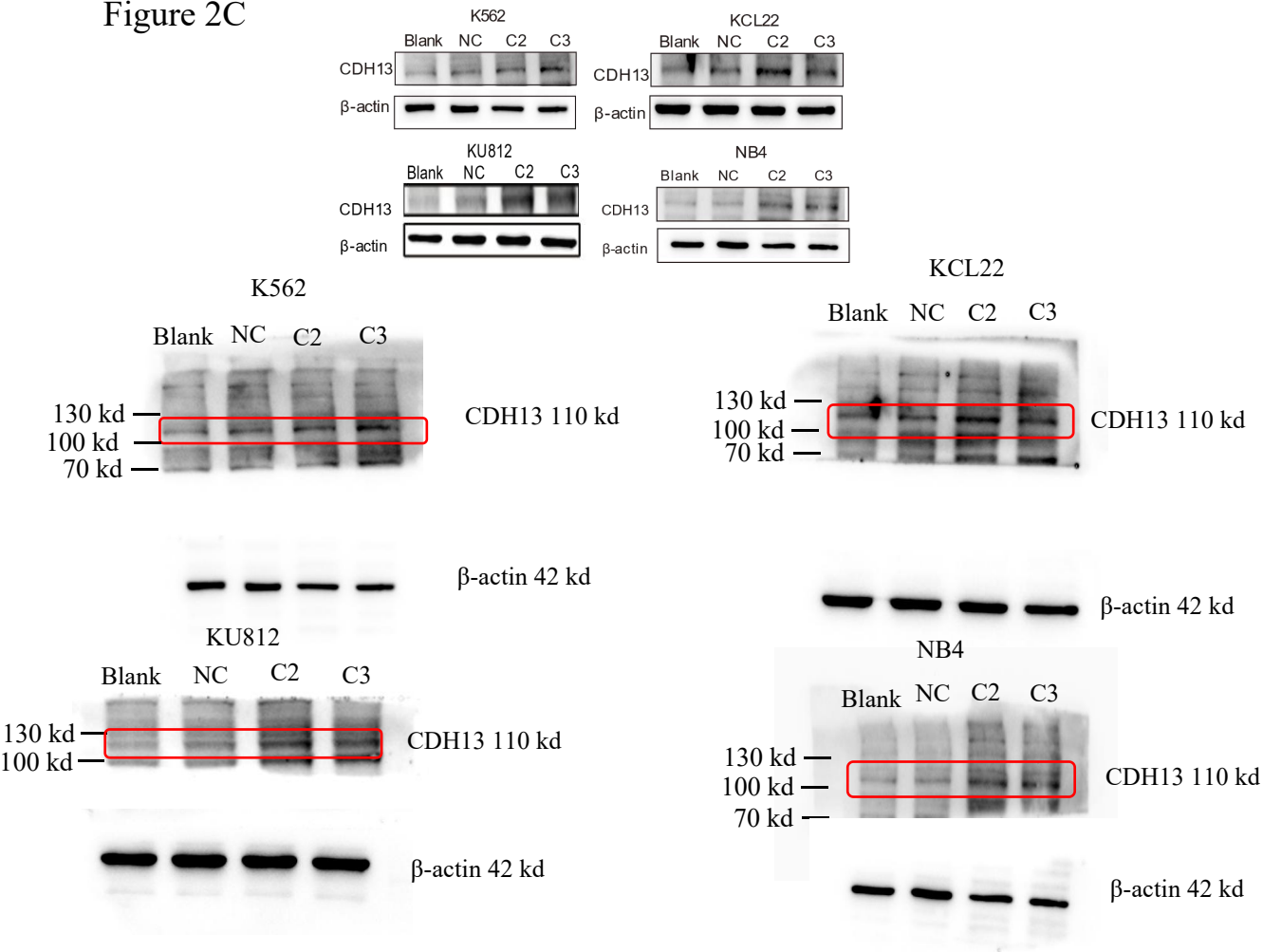

Figure 3D

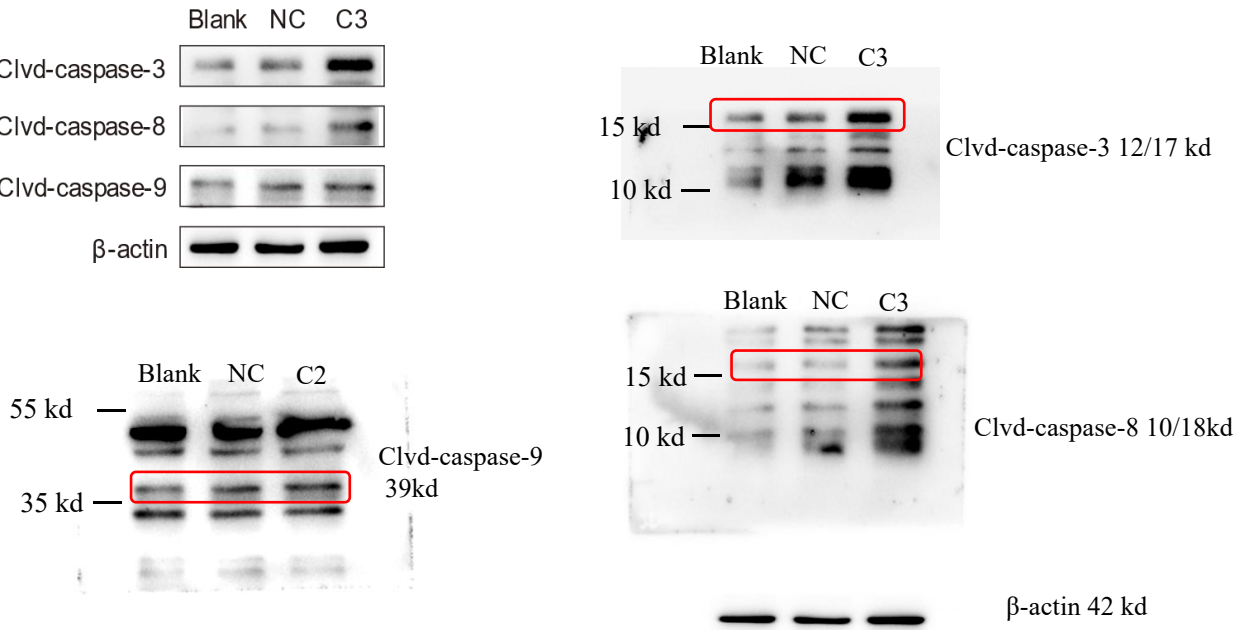

Figure 4A,B

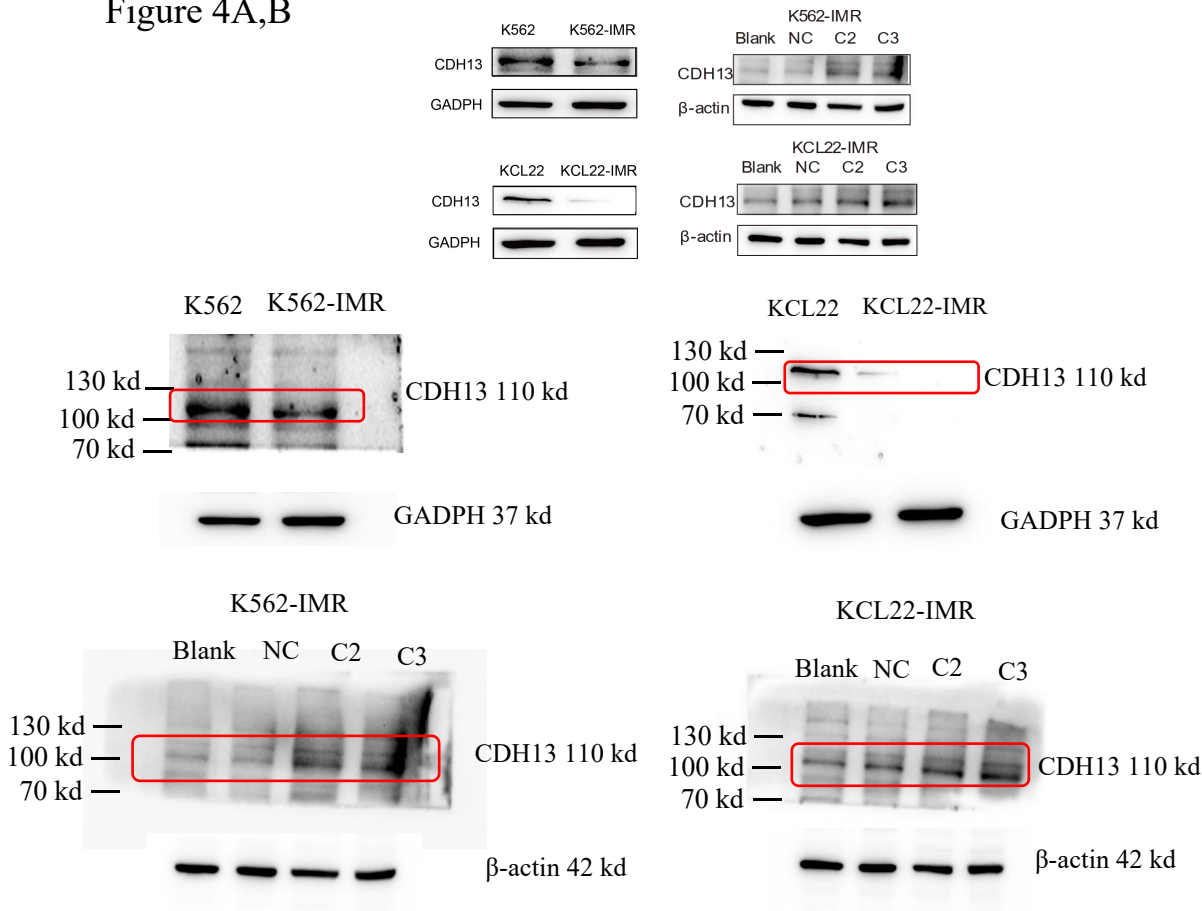

Figure 4E

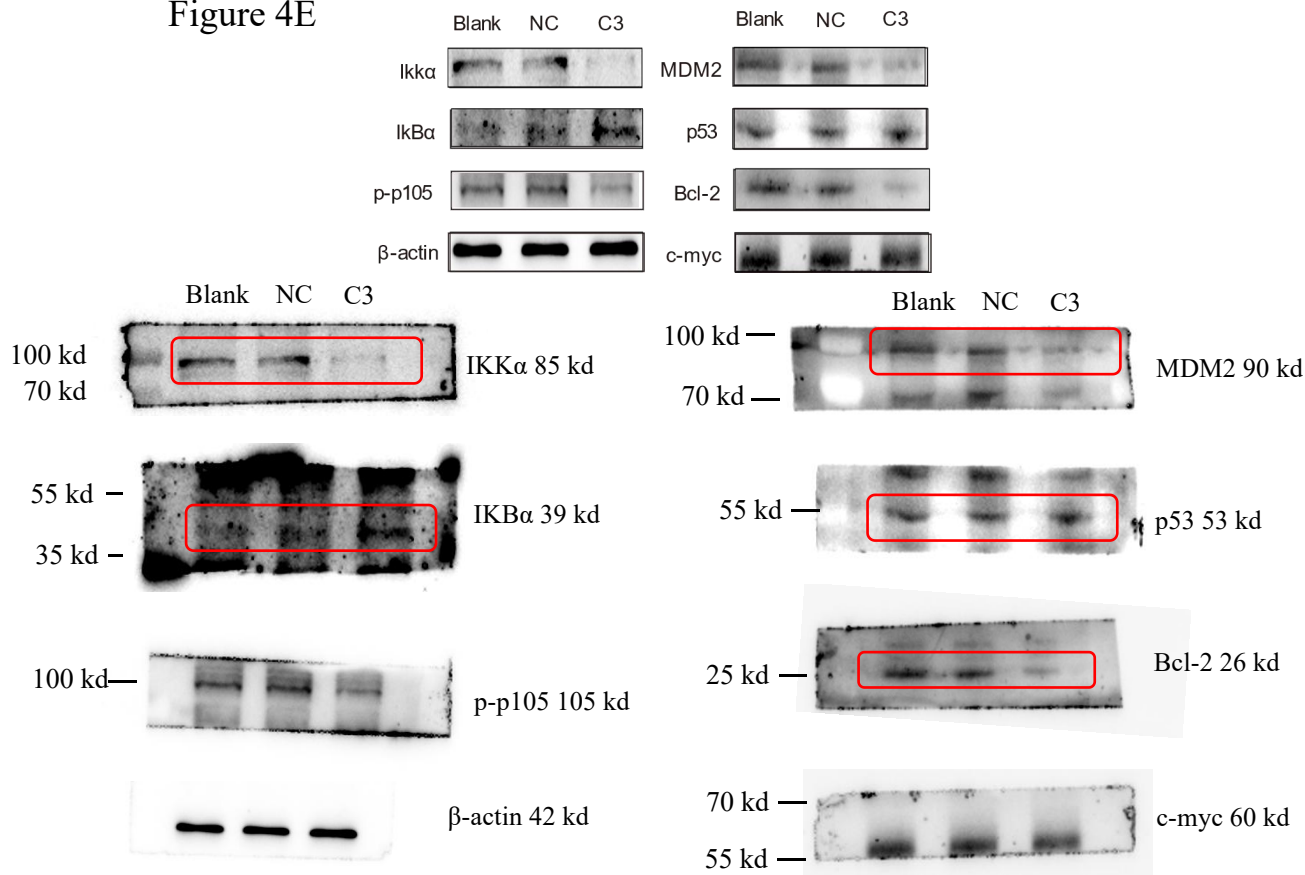

Figure S2

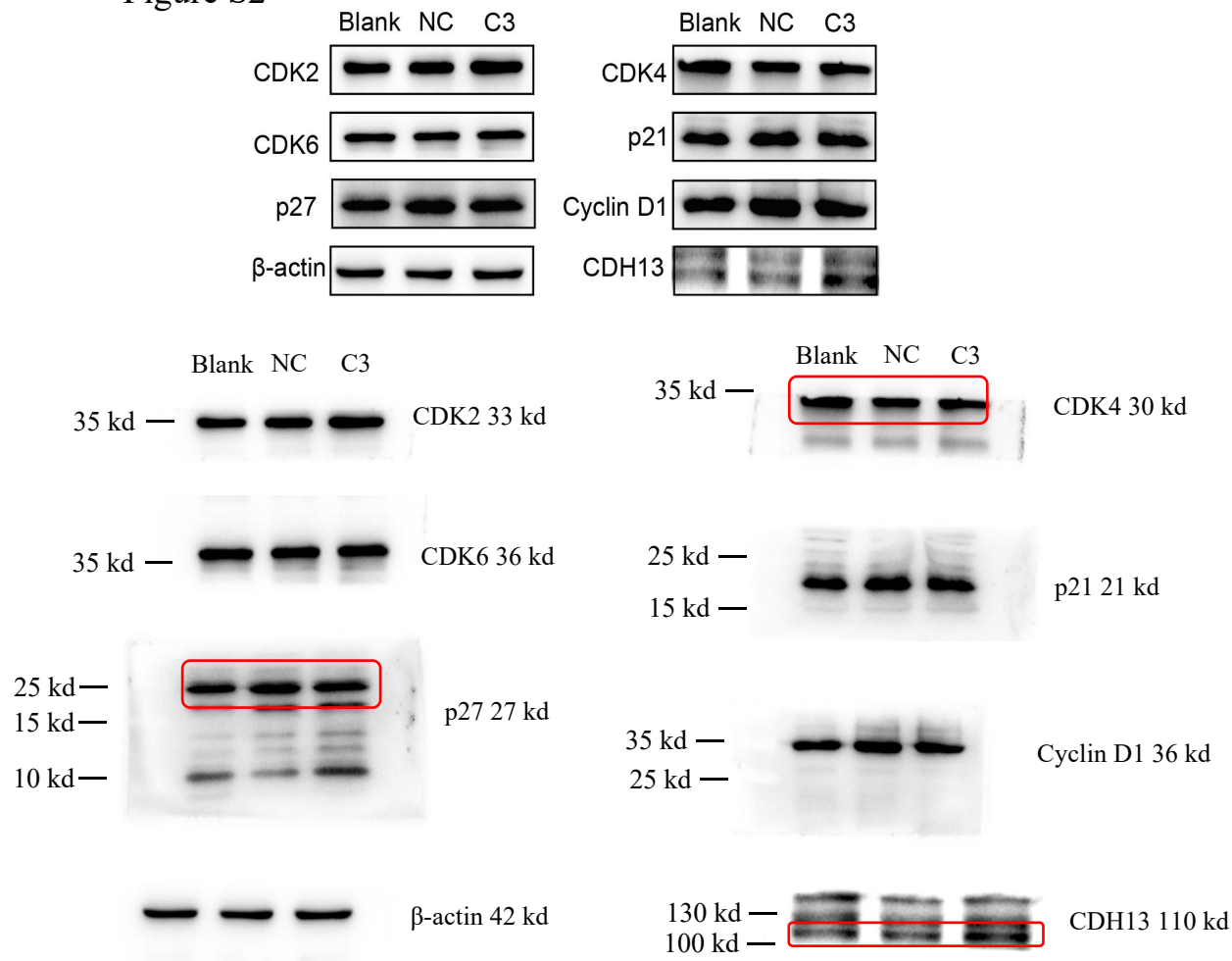

Figure S5

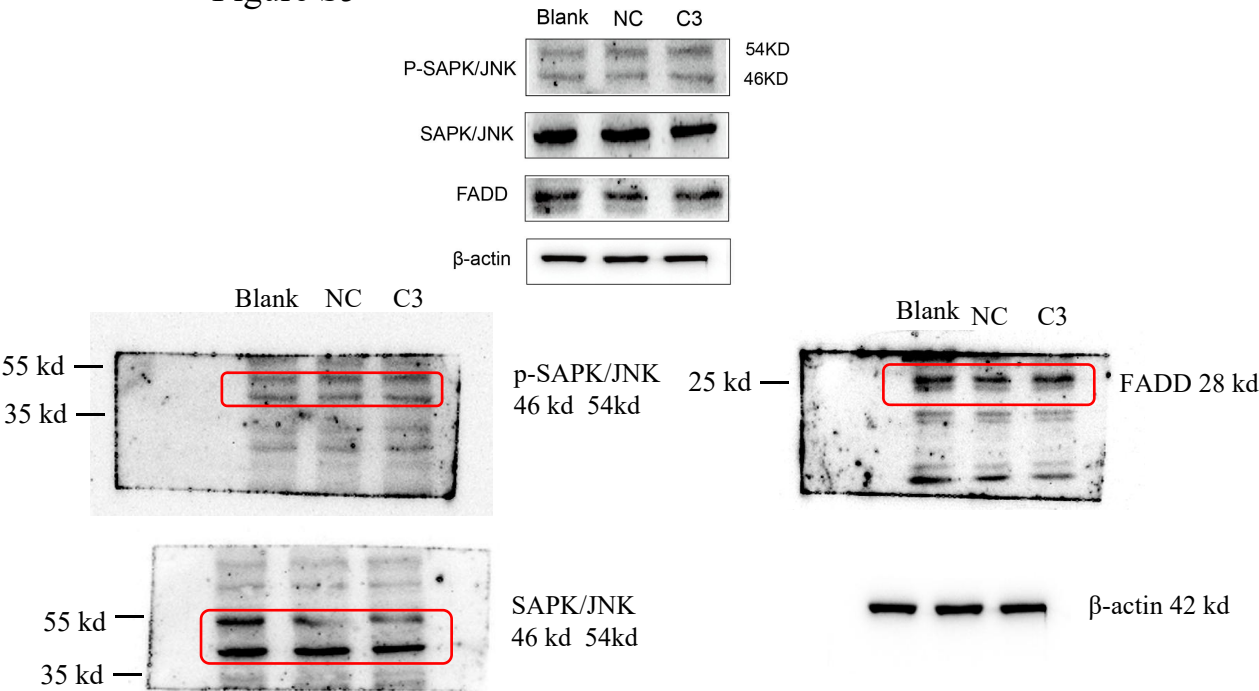

Supplement: Supplementary file 2 — Original Data [file 41419_2024_7006_MOESM2_ESM.pdf]
